# Survival Without Neurological Sequelae Following Massive Pulmonary Embolism and Prolonged Cardiac Arrest

**DOI:** 10.7759/cureus.71898

**Published:** 2024-10-19

**Authors:** Frédéric Paciorkowski, Wael Nahban, Jean-Marie Jacques

**Affiliations:** 1 Emergency Department, Epicura Hospital, Hornu, BEL; 2 Intensive Care Department, Epicura Hospital, Hornu, BEL

**Keywords:** capnography, chain of survival, neurologic recovery, out of hospital cardiac arrest, va-ecmo

## Abstract

We present the case of a 17-year-old female who survived cardiac arrest caused by a massive pulmonary embolism leading to obstructive shock. Despite a prolonged low-flow period of 193 minutes, she fully recovered without apparent neurological damage, thanks to immediate cardiopulmonary resuscitation (CPR) initiated by her father, timely intervention by paramedics and medical teams, and the use of veno-arterial extracorporeal membrane oxygenation (VA-ECMO). Key factors in her recovery included the absence of a no-flow period, maintenance of end-tidal CO_2_ (ETCO_2_) levels above 25-30 mmHg during resuscitation, and the vital role of VA-ECMO in supporting organ perfusion. This case highlights the importance of prompt bystander intervention, continuous ETCO_2_ monitoring during CPR, and the integration of advanced circulatory support measures in post-cardiac arrest care. Additionally, it underscores the potential for favorable neurological outcomes even after extended resuscitation, provided there is adequate cerebral perfusion and timely mechanical support. The report emphasizes the need for public education on basic life support (BLS).

## Introduction

Cardiac arrest caused by massive pulmonary embolism is a life-threatening condition with high mortality. Early recognition, prompt intervention, and advanced circulatory support play crucial roles in determining the outcome. Despite the prolonged low-flow states, neurologically intact survival remains possible with timely mechanical assistance, such as veno-arterial extracorporeal membrane oxygenation (VA-ECMO) [1-3], and the continuous assessment of circulatory effectiveness through tools like end-tidal CO_2_ (ETCO_2_) monitoring [4]. In this report, we present the case of a 17-year-old female who experienced a cardiac arrest due to a massive pulmonary embolism. Despite an exceptionally long resuscitation period, she recovered without neurological sequelae, underscoring the critical importance of immediate CPR, continuous ETCO_2_ monitoring, and VA-ECMO in post-cardiac arrest management. This case also highlights the importance of public education in basic life support (BLS) and the need for advanced diagnostic and therapeutic interventions in pre-hospital settings [5,6].

## Case presentation

A 17-year-old woman, while watching television and sitting on a sofa, experienced a sudden loss of consciousness following complaints of lightheadedness. Notably, she did not report any chest pain at the time or in the days prior. She had two episodes of transient loss of consciousness the previous day and the day before. Her medical history was unremarkable, except for the use of oral contraception. She was a non-smoker and allergic to penicillin. Her mother had a history of four episodes of pulmonary embolism without a thrombophilia workup.

On the day of the incident, her father, who was sitting next to her, immediately started cardiopulmonary resuscitation (CPR), ensuring no period of no-flow. Paramedics arrived nine minutes after the call and continued CPR, deploying an automated external defibrillator (AED), which detected no shockable rhythm. A brief interruption of CPR for one minute occurred due to the appearance of a pulse.

The advanced medical team arrived 22 minutes post-call. Pulseless electrical activity (PEA) was noted on the monitor, with a very weak pulse and no blood pressure. The patient was intubated, and CPR was continued with a Lucas device installed 26 minutes post-call. A total of 6 mg of adrenaline was administered along with continuous norepinephrine, and 5000 IU of heparin was also given. ETCO_2_ levels were extremely low without massage, rising to 40 ppm when massage continued. Due to the absence of ultrasound equipment and fibrinolytics, the patient was prepared for transport to an intensive care unit (ICU).

**Figure 1:**
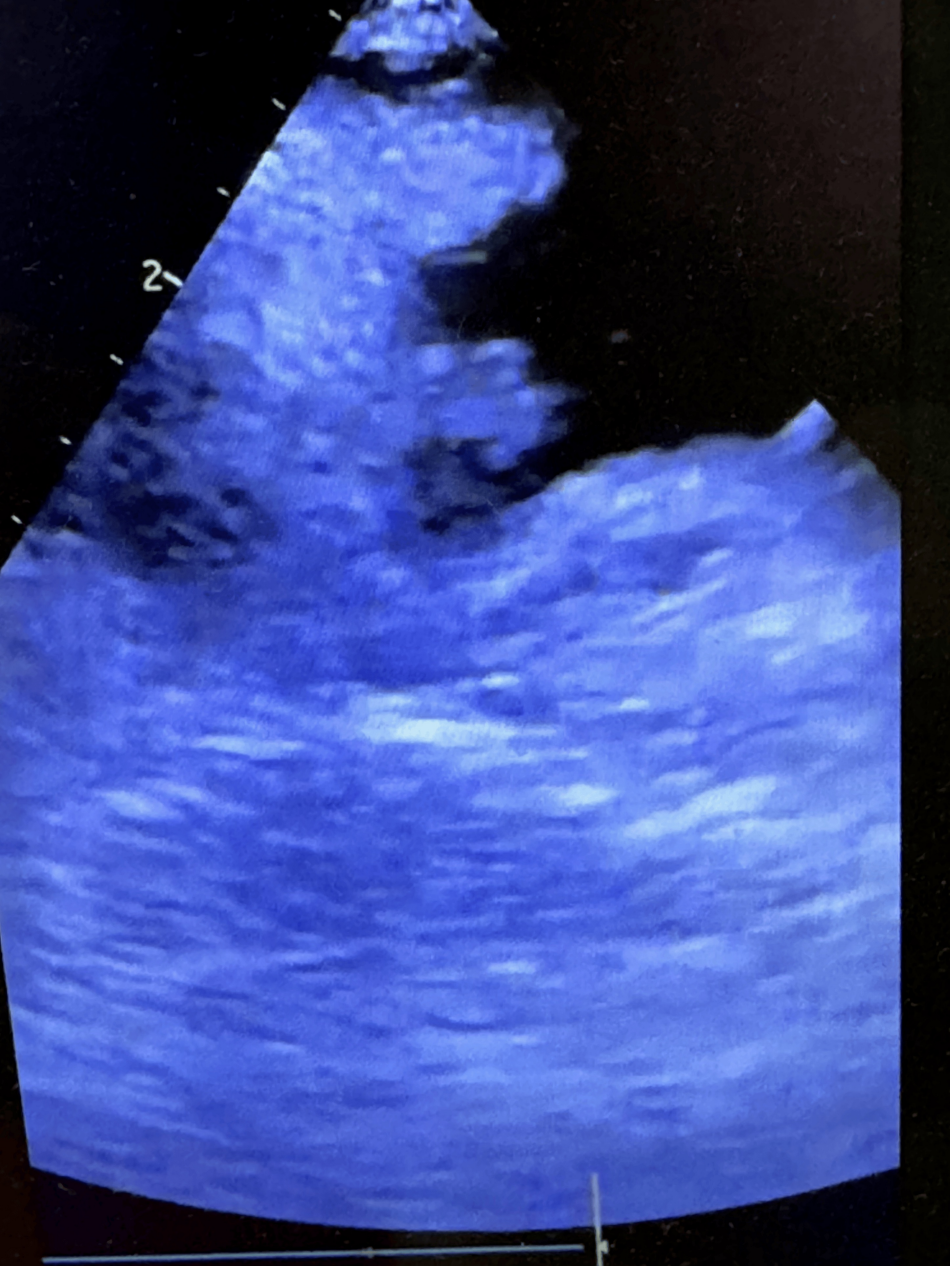
Transesophageal ultrasound showing an endocavitary thrombus

After a total of 153 minutes from the initial call, the patient reached the hospital. The first blood gas shows a pH of 6.98, a PaO_2_ of 63 mmHg, a pCO_2_ of 45 mmHg, a bicarbonate of 10.6 mmol/L, and a lactate of 6.9 mmol/L. Given the absence of administration of a thrombolytic agent, a VA-ECMO device was placed via femoral veins and became functional 193 minutes after the call, allowing for the cessation of CPR. Transesophageal echocardiography revealed a right atrial thrombus, right ventricular dilation, and severe left ventricular systolic dysfunction (Figure 1).

During her hospital stay, the patient developed transient acute kidney injury (AKI), biological pancreatitis, a urinary tract infection with *Escherichia coli*, and respiratory tract contamination with methicillin-sensitive *Staphylococcus aureus* (MSSA). Noradrenaline could be weaned in 48 hours, and transesophageal echocardiography shows the thrombus clearing and normalization of left ventricular function. She was weaned off mechanical support after four days. An embolectomy was required due to right femoral arterial thrombosis during catheter removal, with no further complications. She was successfully weaned off the ventilator and fully recovered without any apparent neurological sequelae. She was discharged from the ICU after five days and was able to leave the hospital four days later, without the need for rehabilitation.

## Discussion

This case highlights the importance of minimizing the no-flow period, emphasizing the irreplaceable contribution of active intervention by the first responders with the immediate initiation of CPR. Public education in BLS, starting from school, remains a major public health issue. The coordination between the various responders was optimal, ensuring a seamless chain of survival during an extended period of care.

An extremely prolonged low-flow period does not seem prohibitive for the preservation of higher cerebral functions, with contributions from intrinsic perfusion and CPR. The Lucas device was effective in providing necessary support over a very long period (167 minutes). The insufficiency of the intrinsic perfusion generated by the patient, related to both obstruction and left ventricular dysfunction, was demonstrated by the ETCO_2_ measurements. ETCO_2_ levels dropped during interruptions in CPR and were restored to 40 mmHg when CPR was resumed. This observation underscores the importance of ETCO_2_ monitoring during circulatory assessment in cardiac arrest.

The finding that an ETCO_2_ level greater than 10 mmHg indicates the return of spontaneous rhythm and circulation is crucial [7]. Moreover, the observation that maintaining ETCO_2_ at higher levels (>25 mmHg) has been associated with the absence of neurological sequelae suggests that continuing CPR in cases of cardiac arrest to achieve ETCO_2 _levels above 25 mmHg may be linked to a better neurological prognosis [8-11].

VA-ECMO played a critical role in preserving the patient’s higher cognitive functions in this context of mixed shock. Its use is beneficial in post-cardiac arrest dysfunctions when there is a real hope of recovering satisfactory function, either in addition to or as a replacement for hypoxemic amines.

## Conclusions

This young woman survived a cardiac arrest caused by a massive pulmonary embolism with obstructive shock. Despite a prolonged low-flow period of 193 minutes, the absence of a no-flow period, maintenance of ETCO_2_ levels greater than 25-30 mmHg, and timely intervention with veno-arterial mechanical assistance contributed to her recovery without any apparent neurological damage.

## References

[1] Giraud R, Laurencet M, Assouline B, De Charrière A, Banfi C, Bendjelid K (2021). Can VA-ECMO be used as an adequate treatment in massive pulmonary embolism?. J Clin Med.

[2] Kim YS, Choi W, Hwang J (2017). Resuscitation of prolonged cardiac arrest from massive pulmonary embolism by extracorporeal membrane oxygenation. Eur J Cardiothorac Surg.

[3] Hsin T, Chun F W, Tao HL (2014). Ultra-long cardiopumorary resuscitation with thrombolytic therapy for un sudden cardiact arrest patient with pulmonary embolism. Am J Emerg Med.

[4] Nicholson TC, Paiva EF (2020). Uses and pitfalls of measurement of end-tidal carbon dioxide during cardiac arrest. Curr Opin Crit Care.

[5] Sandroni C, De Santis P, D'Arrigo S (2018). Capnography during cardiac arrest. Resuscitation.

[6] Paiva EF, Paxton JH, O'Neil BJ (2018). The use of end-tidal carbon dioxide (ETCO2) measurement to guide management of cardiact arrest: a systematic review. Resuscitation.

[7] Levine RL, Wayne MA, Miller CC (1997). End-tidal carbon dioxide and outcome of out-of-hospital cardiac arrest. N Engl J Med.

[8] Poon KM, Lui CT, Tsui KL (2016). Prognostication of out-of-hospital cardiac arrest patients by 3-min end-tidal capnometry level in emergency department. Resuscitation.

[9] Touma O, Davies M (2013). The prognostic value of end tidal carbon dioxide during cardiac arrest: a systematic review. Resuscitation.

[10] Algahtani H, Azzam M, Albanna AS, Shirah B (2018). Neurological recovery from multiple cardiac arrests due to acute massive pulmonary embolism managed by cardiopulmonary resuscitation and extracorporeal membrane oxygenation. Cardiovasc Revasc Med.

[11] Sheak K, Wiebe DJ (2015). Quantitative relationship between end-tidal carbon dioxide and CPR quality during both in-hospital and out-of-hospital cardiac arrest. Resuscitation.

